# Hypersensitivity Reactions to Platinum Agents and Taxanes

**DOI:** 10.1007/s12016-021-08877-y

**Published:** 2021-08-02

**Authors:** Lulu R. Tsao, Fernanda D. Young, Iris M. Otani, Mariana C. Castells

**Affiliations:** 1 UCSF Department of Medicine, Division of Pulmonary, Critical Care, Allergy and Sleep Medicine, San Francisco, CA USA; 2 Brigham and Women’s Hospital, Department of Medicine, Division of Allergy and Clinical Immunology, Boston, MA USA

**Keywords:** Chemotherapy, Hypersensitivity, Drug allergy, Desensitization, Platinum agent, Taxane

## Abstract

Hypersensitivity reactions (HSRs) to chemotherapy agents can present a serious challenge to treating patients with preferred or first-line therapies. Allergic reactions through an immunologic mechanism have been established for platinum and taxane agents, which are used to treat a wide variety of cancers including gynecologic cancers. Platin HSRs typically occur after multiple cycles of chemotherapy, reflecting the development of drug IgE sensitization, while taxane HSRs often occur on first or second exposure. Despite observed differences between platin and taxane HSRs, drug desensitization has been an effective method to reintroduce both chemotherapeutic agents safely. Skin testing is the primary diagnostic tool used to risk-stratify patients after initial HSRs, with more widespread use for platinum agents than taxanes. Different practices exist around the use of skin testing, drug challenge, and choice of desensitization protocol. Here, we review the epidemiology, mechanism, and clinical presentation of HSRs to platinum and taxane agents, as well as key controversies in their evaluation and management.

## Introduction

More than 20 years ago, platinum agents and paclitaxel became the standard first-line regimens for treatment of ovarian cancer based on clinical trial data from the Gynecologic Oncology Group and European investigators [1]. Their increased use was accompanied by a relatively high prevalence of hypersensitivity reactions (HSRs) in women requiring repeated cycles of chemotherapy. Rapid drug desensitization (RDD) has allowed patients to continue receiving first-line treatments and avoid unnecessary switches to second- or third-line therapies that might be less effective or more toxic. Since the early 2000s, many institutions have implemented protocols for evaluation and treatment of patients with HSRs to chemotherapy agents. Along the way, there has been significant evolution in our understanding of both IgE-mediated and non-IgE-mediated reactions to these drugs. Areas of active research include optimal risk stratification based on patient history, skin testing, and biomarkers, as well as the safety and efficacy of various desensitization protocols and strategies for delabeling.

## Clinical Presentation

### Platinum Agents

Carboplatin and cisplatin are commonly used for ovarian, lung, and head and neck cancers (Table 1) [2]. Carboplatin-based regimens are frequently recommended for women with platinum-sensitive recurrent ovarian cancer [3, 4]. Oxaliplatin is commonly used in colorectal cancer regimens as first-line or adjuvant treatment for metastatic disease [5].

**Table 1:** Characteristics of immediate HSRs to platinum and taxane agents

**Agent**	**Incidence**	**Common uses**	**Timing**	**HSR characteristics**
Carboplatin [10, 13]	8–16% in gynecologic cancers [6–8] Pediatric: 9% in solid tumors [66], 21–42% in low-grade glioma [67, 68]	• Ovarian cancer, initial treatment (standard 6 cycles) and recurrence • Lung cancer • Head and neck SCC	Peak rate of HSR occurs with cycle 8 or 9 (2nd or 3rd cycle after restarting treatment for recurrence) • 1% between cycles 1–6 • 27% for cycle 7 or more • 46% for cycle 15 or more	Flushing, pruritis, urticaria Approximately 50% of HSRs are moderately severe with diffuse erythroderma, wheezing, facial swelling, nausea/vomiting, diarrhea, dyspnea, hypotension, or anaphylaxis
Cisplatin [2, 3, 69]	5–20%	• Gynecologic malignancy, e.g. ovarian, uterine, and endometrial carcinoma • Lung cancer • Head and neck SCC	Increases with number of cycles and with concomitant radiation; higher rate after cycle 6	Urticaria, pruritis, respiratory distress, hypotension
Oxaliplatin [5, 10, 14, 31]	7–24% Severe HSR in 0.5–2%	Gastrointestinal malignancy, especially colon cancer (usually in combination with leucovorin and fluorouracil, or FOLFOX/FOLFIRI)	Increases with number of cycles; up to 20% after cycle 6	Flushing, pruritis, urticaria, palmar erythema, angioedema, hypertension, hypotension, dyspnea, chest tightness, cough, throat tightness, nausea, diarrhea Less commonly, cytokine release reactions with fever, chills, rigors, back pain Increased neurological (tingling, dizziness) and systemic symptoms relative to carboplatin and cisplatin Rarely, thrombocytopenia, hemolytic anemia, and bleeding can also occur during HSRs [14, 31]
Paclitaxel Albumin-bound [15]	4% without premedication	• Breast cancer • Ovarian cancer • Non-small cell lung cancer • Head and neck SCC • AIDS-related Kaposi’s sarcoma (Cremophor-bound paclitaxel) • Prostate cancer (docetaxel) • Gastric adenocarcinoma (docetaxel) • Pancreatic adenocarcinoma (albumin-bound paclitaxel)	On first exposure during cycle 1 or 2	Flushing, chest pain, back pain, abdominal pain, respiratory symptoms (dyspnea, chest tightness, wheezing, throat tightness), gastrointestinal symptoms (nausea, vomiting, diarrhea), hypertension, hypotension, sense of impending doom Fluid retention also seen with docetaxel
Paclitaxel Cremophor-bound [10, 15]	10% despite premedication in gynecologic cancers [16, 17]			
Docetaxel [15]	5% despite premedication			
Cabazitaxel [15, 70]	0% in metastatic castration-resistant prostate cancer [71, 72] 6% in phase II study of metastatic breast cancer [70, 73]	Hormone-resistant metastatic prostate cancer in patients previously treated with docetaxel		

*SCC* squamous cell carcinoma

Reported incidence, indications, and clinical presentations of immediate HSRs to platinum agents are shown in Table 1. The incidence of carboplatin HSRs has been best described in gynecologic cancer, especially ovarian cancer, at rates of 8–16% [6–8]. Incidence among patients with other types of cancers is less well known. At one center, carboplatin HSR occurred in 7.9% of ovarian cancer patients and 2.6% of all cancer patients, including lung, head and neck, other gynecologic, and breast cancers [9]. However, when evaluated by total lifetime dose or number of cycles, incidence was similar regardless of cancer type [9].

The main risk factor for platin HSR is prior treatment with these therapies [3]. The rate of carboplatin HSR increases from 1% during the first six cycles to 27% after 7 doses, and the peak rate occurs with cycle 8 or 9, which usually corresponds to the second or third cycle after restarting treatment for recurrent disease [10]. Similar patterns have been seen with cisplatin and oxaliplatin [2, 3]. Increased rates have also been observed in women with BRCA mutations [11] and with certain chemotherapy regimens. For example, in the CALYPSO study, women treated with carboplatin/paclitaxel had a higher incidence of HSR compared to carboplatin/doxorubicin regimens [12].

**Fig. 1:**
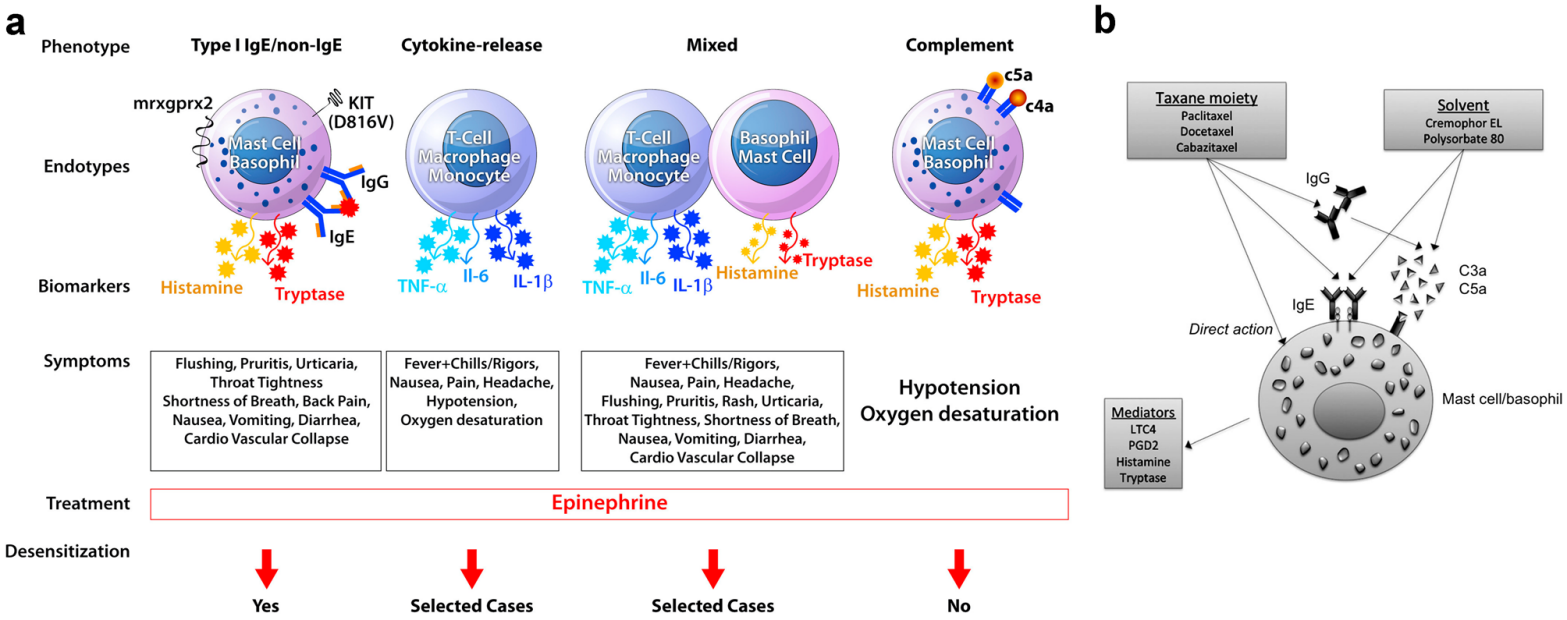
Mechanisms of immediate HSRs to platins and taxanes. Phenotypes of platin HSRs include type I reactions, cytokine release reactions, and mixed reactions, with the most heterogeneity seen with oxaliplatin (**A**). Taxanes may cause mast cell and/or basophil activation through IgE-mediated mechanisms, direct action on basophils, or IgG-mediated mechanisms that cause complement activation and release of anaphylatoxins (C3a, C5a) (**A**, **B**). Solvents for taxanes, such as Cremophor EL (paclitaxel) and polysorbate 80 (docetaxel), may also activate mast cells through an IgE-mediated mechanism or direct complement activation. Biomarkers include tryptase, histamine, leukotrienes, and prostaglandins in type I reactions and IL-6, TNF-α, and IL-1ß in cytokine release or mixed reactions (**A**, **B**). Desensitization is indicated for type I reactions and selected cases of cytokine release and mixed reactions, but not in direct mast cell/basophil activation (**A**). *LTC4* leukotriene C4, *PGD2* prostaglandin D2. Reproduced from Fig. 1 in Castells [65] and Fig. 3 in Picard and Castells [15] with permission

Platinum agents can cause type I reactions, cytokine release reactions, and mixed reactions, and mechanisms for each phenotype are shown in Fig. 1A. Evidence supporting an IgE-mediated mechanism for HSRs to platinum agents was first described in refinery workers exposed to platinum salts and subsequently with the detection of carboplatin-specific IgE [13]. Most reactions are immediate and occur during or within hours after infusion [3, 4]. For oxaliplatin specifically, HSRs are more heterogeneous and include cytokine release reactions presenting with fevers, chills, rigors, headache, chest pain, and/or back pain along with elevated levels of IL-6 and TNF-α [5]. Oxaliplatin can also induce mixed reactions with symptoms of both type 1 hypersensitivity and cytokine release reactions [5]. This variability has motivated efforts to endophenotype oxaliplatin reactions to better predict outcomes with desensitization [5]. In contrast to carboplatin and cisplatin, cases of immune-mediated hemolytic anemia and thrombocytopenia complicated by bleeding have also been reported for oxaliplatin [5, 14]. Delayed rashes have been reported hours to days after carboplatin infusion, ranging in severity from mild reactions to skin desquamation [13]. There have not been any reports of Stevens-Johnson syndrome/toxic epidermal necrolysis (SJS/TEN), erythema multiforme, or serum sickness with carboplatin [10].

### Taxanes

Taxane agents are used in the treatment of breast, gynecologic, prostate, head and neck, and lung cancers, and include paclitaxel, docetaxel, nab-paclitaxel, and cabazitaxel (Table 1) [15]. Paclitaxel (Taxol) was originally isolated from the bark of the Pacific yew tree, while docetaxel (Taxotere) was made from a semisynthetic process [15].

Similar to carboplatin, the incidence of HSR to paclitaxel has been best studied in gynecologic cancer [16, 17]. Initial studies found rates of immediate HSRs of up to 50% with paclitaxel and docetaxel infusions, resulting in the routine use of antihistamine and steroid premedication [15, 18, 19]. Incidence rates, indications, and clinical presentations of immediate HSRs for taxanes are presented in Table 1. Immediate HSRs to paclitaxel and docetaxel occur in approximately 10% of patients despite premedication and are severe in 1% [10, 18]. They commonly occur within minutes during the first or second lifetime exposure, with symptoms such as flushing, dyspnea, throat tightness, hypotension, as well as more atypical symptoms like chest or back pain [18, 19]. Delayed rashes have also been reported hours to weeks after infusion [3, 10]. Severe reactions such as SJS/TEN, acute interstitial pneumonitis, and subacute cutaneous lupus erythematous have been described in case reports with paclitaxel, docetaxel, and nab-paclitaxel [10, 15].

Potential mechanisms for immediate taxane HSRs are shown in Fig. 1B. Due to the timing of reaction with first or second exposure, the mechanism has been thought to be non-IgE-mediated in some cases and possibly related to infusion solvents, although initial data indicated that a majority of patients were allergic and cross-reactivity with tree pollen allergens was suggested [20]. Cremophor EL in paclitaxel and polysorbate 80 in docetaxel and cabazitaxel are solubilizing and emulsifying agents that can cause complement activation with anaphylatoxin production and mast cell activation in vitro [15, 18]. Polysorbate 80 may also cause direct mast cell activation via peroxide formation [21]. Data supporting the role of Cremophor includes the decreased rate of immediate HSRs with nanoparticle albumin-bound paclitaxel (nab-paclitaxel), with no reactions seen in phase I, II, or III studies despite omitting premedication [22–24]. Nevertheless, severe immediate HSRs have still been reported with nab-paclitaxel in post-marketing surveillance [15]. More recently, IgE-mediated mechanisms for at least a subset of taxane HSRs have been suggested based on positive skin test results and immunoblot assays [15]. One postulated mechanism for the occurrence of IgE-mediated taxane HSRs during initial cycles is that patients living in parts of the world with yew trees may be sensitized by pollen exposure [15]. Paclitaxel has also been isolated from hazelnuts [25], and hypersensitivity reactions to both paclitaxel and hazelnut ingestion have been described [26]. Other suggested mechanisms include histamine release through a direct effect of paclitaxel on basophils [15].

## Classification and Grading of HSRs

Multiple classification systems exist to describe HSR severity. This has important implications as initial HSR severity has been used to guide treatment decisions such as rechallenge and choice of desensitization protocol [27, 28]. It also contributes to the challenge of comparing and contrasting published findings that use different grading systems.

The most commonly used classifications include Brown’s grading system and the Common Terminology Criteria for Adverse Events (CTCAE) (Table 2). The Brown classification incorporates specific elements of HSRs and severity of organ involvement used by allergists, while CTCAE focuses on the duration of reaction and interventions required. The CTCAE is a National Cancer Institute (NCI) classification system for chemotherapy adverse event reporting and grades infusion reactions based on severity [29]. The Brown grading system was originally created for a wide range of HSRs and is not specific to chemotherapy agents or even drug allergy. It is based on the association of specific symptoms with severe reactions, such as hypotension and hypoxia, among 1149 patients presenting to an ED with HSRs [30]. When applied to chemotherapy agents, Brown grade 1 (mild) reactions would be limited to the skin or involve a single organ system, grade 2 (moderate) reactions involve 2 or more organ systems without a significant decrease in blood pressure or oxygen saturation, and grade 3 (severe) reactions include vital sign changes such as hypotension, oxygen desaturation, and cardiovascular collapse [31, 32].

**Table 2:** Comparison of grading systems

**Grading system and application**	**Grade/severity and definitions**			
CTCAE v5.0* for infusion-related reactions [29]	**Grade 1** Mild transient reaction Infusion interruption not indicated Intervention not indicated	**Grade 2** Therapy or **infusion interruption indicated** but **responds promptly** **to symptomatic treatment** (e.g., antihistamines, NSAIDs, opioids, intravenous fluids)	**Grade 3** Prolonged (e.g., **not rapidly responsive** to symptomatic medication and/or brief interruption of infusion) **Recurrence** of symptoms following initial improvement **Hospitalization** indicated for other clinical sequelae	**Grade 4** **Life-threatening** consequences Urgent intervention indicated
Brown grading system for general HSRs [30]	**Grade 1 (mild)** **Skin and subcutaneous tissues only**, e.g., generalized erythema, urticaria, periorbital edema, or angioedema	**Grade 2 (moderate)** Features suggesting respiratory, cardiovascular, or gastrointestinal **organ involvement**, e.g., dyspnea, stridor, wheeze, nausea, vomiting, dizziness (presyncope), diaphoresis, chest or throat tightness, or abdominal pain		**Grade 3 (severe)** Hypoxia, hypotension, or neurological **organ compromise**, i.e., cyanosis or SpO2 ≤ 92% at any stage, hypotension (SBP < 90 mmHg in adults), confusion, collapse, LOC, or incontinence
Brown grading system, example of adaptation for taxane HSRs [32]	**Grade 1 (mild)** Symptoms limited to the skin (e.g., flushing) or involve a **single organ/system** and are mild (e.g., mild back pain)	**Grade 2 (moderate)** Symptoms involve **at least 2 organs/systems** (e.g., flushing and dyspnea), but there is no significant decrease in blood pressure or oxygen saturation		**Grade 3 (severe)** Symptoms typically involve at least 2 organs/systems, and there is a **significant decrease in blood pressure** (systolic ≤ 90 mm Hg and/or syncope) **and/or oxygen saturation** (≤ 92%)

^*^CTCAE also includes grade 5 which is death

## Role of Skin Testing

Skin testing (ST) is the most widely used diagnostic tool in the evaluation and risk stratification of platin and taxane HSRs, although specific IgE (sIgE), basophil activation test (BAT), and other biomarkers such as soluble FcεRI and total IgE have also been studied [33–35].

### Platinum Agents

Carboplatin skin testing was first developed in the 1990s and has been increasingly used in the evaluation and management of carboplatin HSRs [13]. However, differences in practice remain, with some institutions using serial ST for risk stratification [36, 37] and others not using ST at all [28]. The negative predictive value (NPV) of a single intradermal test for carboplatin has been estimated at 81–92% [13]. Accurate estimates of positive predictive value (PPV) are limited by the ethical issues of challenging patients after a positive ST. However, in one study, 6 of 7 ST-positive patients experienced an HSR with standard infusion, thus providing a PPV of 86% [38]. For oxaliplatin, a recent study did not find any association between skin testing and breakthrough reactions [5], whereas a separate study of 74 oxaliplatin-reactive patients reported sensitivity and specificity of 57.5% and 91.7%, respectively [33]. There is a need for additional studies and rigorous study designs to assess predictive values of ST.

#### Concentrations

Reported nonirritating concentrations for platinum skin testing are shown in Table 3. The intradermal test (IDT) is needed to achieve adequate sensitivity, as 86.4% of positive carboplatin ST are identified by intradermal testing [13]. The concentration used for testing affects the predictive value of ST. Lower concentrations of 1–5 mg/mL have resulted in positive ST for 41–75% of patients, whereas a final intradermal concentration of 10 mg/mL has resulted in positive ST for 81–88% [13]. However, higher concentrations (10 mg/mL) of carboplatin and oxaliplatin are generally avoided as they can cause irritation and are associated with a risk of skin necrosis and subsequent scarring [4, 10, 36].

**Table 3:** Nonirritating concentrations for platinum agent skin testing

**Agent**	**SPT dilutions (mg/mL)**	**IDT dilutions (mg/mL)**^a^
**Carboplatin**	10	0.1
		1
		5^b^
**Oxaliplatin**	5	0.05
		0.5
		5
**Cisplatin**	1	0.01
		0.1
		1

Variations exist: the European Academy of Allergy and Clinical Immunology (EAACI) recommends concentrations of 10 and 1 mg/mL for carboplatin SPT and IDT and 1 and 0.1 mg/mL for oxaliplatin and cisplatin SPT and IDT [73]

*SPT* skin prick test, *IDT* intradermal test

^a^For intradermal tests, 0.02–0.03 mL is used

^b^Reported concentrations for the last IDT step include 3, 5, and 10 mg/mL; 10 mg/mL has been reported to cause local skin necrosis and is therefore not recommended

#### Timing

The length of time between ST and prior carboplatin exposure or HSR is also important in the interpretation. Based on data from hymenoptera venom skin testing data and the risk of false negatives due to anergy, it is generally recommended that skin testing be performed at least 4 to 6 weeks after the initial HSR [2, 13]. Some recent studies have reported using a window of 2 weeks [5, 39]. However, false negatives may result. In one of these studies, 5/37 (13.5%) ST-negative patients who had been tested within 10 days of an immediate HSR to platinum agents developed recurrent reactions and subsequently converted to ST-positive [39].

There are also higher rates of false-negative ST if performed more than 6 months after initial carboplatin and oxaliplatin HSR, which suggests waning of ST reactivity over time. These patients may undergo conversion from ST-negative to ST-positive after their first desensitization [4, 14, 36, 37]. In early studies, 12/23 (52%) of carboplatin initial ST-negative patients and 2/21 (10%) of oxaliplatin initial ST-negative patients were found to convert to ST-positive on serial ST [14, 36], and repeat skin testing protocols for risk stratification were developed to identify “ST converters.”

#### Uses and Patient Population

ST has been primarily used as a risk stratification tool to guide choice of desensitization protocol after HSR and identify patients who may be able to eventually transition to faster infusions. Less common uses for ST include identification of sensitized individuals before the occurrence of HSR and identification of patients who can undergo reintroduction via drug provocation testing, which is discussed further in the next section.

Use of serial ST for risk stratification in a platin HSR pathway has been described, where ST-positive patients underwent intermediate 12-step desensitization while ST-negative patients underwent rapid 8-step desensitization, both in the inpatient setting [37]. If patients had 3 negative ST results, they were advanced to 50% infusion rate inpatient and, if no reaction, 50% infusion rate outpatient.

To decrease time and resource needs, a modified intradermal 1-step skin test protocol using only the highest concentration was recently reported for low-risk patients with platin HSRs [40]. These were patients who had tolerated prior intermediate desensitization without an HSR and did not have a history of positive ST. In a pilot study, 8/10 (80%) patients had a negative ID test and 6/10 (60%) were ultimately converted to 50% infusion rate outpatient [40]. Of note, this 1-step protocol has not been studied for use after initial HSR and is not yet recommended in that setting.

### Taxanes

Skin testing has not been as routinely performed for taxanes because the mechanism of HSRs was traditionally thought to be non-IgE-mediated [20, 41]. However, as discussed previously, a subset of patients may react via an IgE-mediated process based on prior sensitization to a cross-reactive pollen from the yew tree [42]. ST has been reported for paclitaxel and, to a more limited extent, docetaxel [19, 32, 33, 39], but not cabazitaxel or nab-paclitaxel [10].

#### Concentrations

Table 4 shows concentrations that have been reported for skin prick and intradermal testing. Taxane ST was the focus of a multicenter study that used skin prick concentrations of 6 mg/mL and 1 mg/mL, and intradermal concentrations of 0.06 mg/mL and 0.01 mg/mL, for paclitaxel and docetaxel, respectively [19]. In 84 patients with a history of immediate HSRs to taxanes, skin prick testing was negative in all patients, whereas intradermal testing was positive in 14 patients (16.7%), 10 to paclitaxel and 4 to docetaxel. ST were negative in 30 control patients exposed to taxanes without HSRs. This study reported a sensitivity of 16.7% (95% CI, 8.7–24.6%) and specificity of 100% for taxane ST. A larger study by Picard et al. reported ST results from 145 patients using skin prick concentrations of 1 mg/mL and 0.4 mg/mL for paclitaxel and docetaxel, respectively, and intradermal concentrations of 0.001 and 0.01 mg/mL for paclitaxel and 0.04 and 0.4 mg/mL for docetaxel. Of 138 paclitaxel ST performed, 5 (4%) were positive on skin prick and 92 (67%) on intradermal test, while none of the 9 docetaxel ST were positive on skin prick and 8 (89%) were positive on intradermal test [32]. In both studies, positive skin tests correlated with grade 3 reactions and cutaneous symptoms, such as flushing [19, 32]. Differences in initial HSR severity or yew sensitization due to geographical area may explain the observed differences in skin test positivity rates [19].

**Table 4:** Reported taxane skin testing protocols

**Reference**	**Agent**	**SPT dilutions (mg/mL)**	**IDT dilutions (mg/mL)**	**No. of patients with ST**	**SPT positive**	**IDT positive**
Picard et al. (2016) [32]	Paclitaxel	1	0.001, 0.01	138	5 (4%)	92 (67%)
	Docetaxel	0.4	0.04, 0.4	9	0	8 (89%)
Pagani et al. (2019) [19]	Paclitaxel	6	0.06	63	0	10 (16%)
	Docetaxel	1	0.01	21	0	4 (19%)

Published skin testing dilutions for skin prick and corresponding intradermal tests from the two largest studies are shown. Protocols have not been compared for different patient characteristics. There were more patients with ovarian cancer and prostate cancer in Picard et al. [32], while breast cancer was the most common cancer in Pagani et al. [19]

#### Timing

ST conversion from negative to positive with taxanes has been infrequently reported compared to platins. In the study of 145 patients who underwent taxane ST, one patient (0.7%) converted [32]. Her initial HSR was a delayed maculopapular rash to paclitaxel and ST was negative. She subsequently tolerated 1 challenge and 3 regular infusions before experiencing an immediate grade 3 HSR and converting to ST-positive on re-evaluation.

#### Uses and Patient Population

One potential use for taxane skin testing is the identification of patients who have infusion reactions but may not need desensitization [42]. This relates to the non-IgE mechanisms postulated for taxane HSRs, as reactions to solvents like Cremophor may be idiosyncratic and not recur on re-exposure. Picard et al. used taxane ST as part of risk stratification to identify 36/164 (22%) patients who could undergo drug challenges and eventually return to standard infusion without need for desensitization [32].

## Role of Drug Provocation Testing

There is debate regarding the role of a diagnostic drug challenge or drug provocation testing (DPT) after an initial HSR to platinum and taxane agents [21].

For platinum agents in particular, prior observational data have shown that there can be high rates of recurrent HSR, including severe HSR, when patients are rechallenged without desensitization. In an early study, symptoms recurred in all 32 patients with carboplatin HSRs who were rechallenged, and 12 patients (38%) discontinued treatment due to severe reactions [7]. Another oncology center rechallenged 14/27 (52%) patients with oxaliplatin HSR, and 4/14 (29%) developed recurrent HSR despite steroid and antihistamine premedication, of which two were CTCAE grades 3–4 [43]. As a result, there has been reluctance to rechallenge patients with platin HSRs. Success rates for rechallenge may be higher for taxane HSRs. A prior observational study found that patients with a history of paclitaxel HSR were more likely to tolerate rechallenge than those with platin HSRs [44]. In addition, as mentioned above, Picard et al. developed a risk stratification approach to rechallenge patients with delayed or grade 1–2 immediate HSRs who had negative ST using a 3-step challenge protocol [32].

Nevertheless, certain institutions in Europe have protocolized the use of DPT for chemotherapy agents and biologics [33, 45–47]. A group reported on 7 years of experience with 102 challenges to taxanes and 93 challenges to platins, which allowed 70 and 43 patients, respectively, to continue treatment without desensitization [45]. These DPTs were performed by administering these agents at standard infusion rates according to manufacturer instructions with only standard premedications [33]. If there were multiple potential culprits (including premedications or concomitant drugs in the regimen, such as leucovorin), DPT was performed for each suspect medication [33, 45]. Severe reactions occurred in up to 7% of the patients who underwent either platin or taxane DPT [45], and the authors emphasize that while DPT can be used as a diagnostic tool with careful patient selection and risk assessment, it is a high-risk procedure that requires specialized resources, monitoring, and expert allergy care [21].

Patients selected for DPT had a history of HSR occurring within 48 h of drug administration and were considered low or medium risk for true HSR. Risk assessment was based on patient-related factors such as comorbidities and acute illness; the drug being administered; prior reaction history, including severity, timing, and need for interventions; and results of ST, sIgE, and other biomarkers if available (e.g., tryptase, IL-6, BAT) [33, 45, 46]. DPT was not performed for patients with delayed reactions or SCARs (such as vasculitis, SJS/TEN, drug-induced hypersensitivity syndrome); previous severe reactions (such as a history of intubation and cardiovascular collapse); high-risk comorbidities (such as uncontrolled asthma, unavoidable use of β‐blockers, critical illness, acute infections); or pregnant patients [33, 45, 48].

A DPT was considered positive if it reproduced the original symptoms or objective findings of HSR [33]. Once symptoms were treated and the patient was asymptomatic, a “restart protocol” was used, beginning at 25% standard rate for 15 min and increased to 50% standard rate until completion [33, 45]. Patients with positive DPT subsequently underwent desensitization. Patients with a negative DPT subsequently underwent standard infusion [33, 45].

The severity of HSRs in patients who underwent DPT in these studies is an important factor that should be examined. In the largest report, the majority of 188 patients with platin HSRs had mild-to-moderate initial HSRs: 42% grade 1, 34% grade 2, and 24% grade 3, using the Brown system [45]. After further risk stratification, 93 of 188 (49%) underwent DPTs, of which 50 (54%) were positive and 7 (14%) of those were grade 3. In line with prior observational findings, more patients tolerated DPTs to taxanes than platins. Of 135 patients referred for taxane HSRs, the initial severity was grade 1 in 20%, grade 2 in 54%, and grade 3 in 26% [45]. One hundred two (76%) were deemed appropriate for DPTs, of which 32 (31%) were positive and 7 (22%) of those were grade 3 reactions.

These initial HSR severity profiles should be compared with other large studies of desensitizations, in which up to 80% (81/101) of initial HSRs were considered severe (characterized by the presence of chest pain, change in blood pressure, dyspnea, oxygen desaturation, or throat tightness), or up to 52% (205/395) were Brown grade 3 [31, 41]. Similarly, in a 2015 report of 92 patients with carboplatin HSRs and 50 patients with oxaliplatin HSRs, the median initial HSR grade was 3 [37]. The majority of patients in these studies had positive ST results [31, 37, 41]. DPT would not be appropriate in these settings, although it may be considered in selected cases, such as in patients with grade 1 reactions and negative ST.

## Desensitization Protocols

Rapid drug desensitization has become the standard of care for patients with platinum and taxane agent HSRs since the early 2000s. Since then, various protocols have been utilized to reintroduce these agents following a reaction. Key differences in desensitization protocols include the number of dilutions and rates of administration.

**Table 5:** Twelve-step desensitization protocol for carboplatin total dose 600 mg

	**Volume (mL)**	**Concentration (mg/mL)**	**Amount infused (mL)**	**Total mg per bag**		
Solution 1	250	0.024	9.38	6		
Solution 2	250	0.24	18.75	60		
Solution 3	250	2.38	250	595.27		
**Step**	**Solution**	**Rate (mL/h)**	**Time (min)**	**Volume infused per step (mL)**	**Dose per step (mg)**	**Cumulative dose (mg)**
1	1	2.5	15	0.63	0.015	0.015
2	1	5	15	1.25	0.03	0.045
3	1	10	15	2.5	0.06	0.11
4	1	20	15	5	0.12	0.23
5	2	5	15	1.25	0.3	0.53
6	2	10	15	2.5	0.6	1.13
7	2	20	15	5	1.2	2.33
8	2	40	15	10	2.4	4.73
9	3	10	15	2.5	5.95	10.68
10	3	20	15	5	11.91	22.58
11	3	40	15	10	23.81	46.39
12	3	80	174.38	232.5	553.61	600
Total time (min) = 339.38 = 5.66 h						

Solution 1 is a 100-fold dilution of the final target concentration; solution 2 is a tenfold dilution of the final target concentration, and the concentration of solution 3 is calculated by subtracting the cumulative dose administered in steps 1–8 from the total target dose and dividing by the bag volume. Values shown are rounded to the nearest 2 decimal places

### Platinum Agents

The most widely accepted desensitization protocol for platinum agents is a 12-step protocol using 3 dilutions (1:100, 1:10, 1:1) with a 2- to 2.5-fold increase between consecutive steps based on in vitro mechanisms of mast cell IgE desensitization (Table 5) [49, 50]. The successful use of a 6-h, 3-bag, 12-step carboplatin desensitization protocol was originally reported in gynecologic oncology patients with the first series of 10 patients who underwent 35 desensitizations, of which 31 (89%) were completed without reaction and the remaining four involved mild cutaneous reactions that did not prevent completion [51]. In 2008, a larger case series of 413 chemotherapy desensitizations using the 12-step protocol was published, including 212 to carboplatin, 12 to cisplatin, and one to oxaliplatin [41]. Two thirds of desensitizations occurred without reaction; another 27% were mild cutaneous reactions requiring only antihistamine, and all breakthrough reactions were less severe than initial HSRs. As most reactions occur during the last step, the addition of a 60 mL/h step between steps 11 and 12 has also been used [2, 10].

**Table 6:** Eight-step desensitization protocol for carboplatin total dose 600 mg

	**Volume (mL)**	**Concentration (mg/mL)**	**Amount infused (mL)**	**Total mg per bag**		
Solution 1	250	0.24	18.75	60		
Solution 2	250	2.38	250	595.5		
**Step**	**Solution**	**Rate (mL/h)**	**Time (min)**	**Volume infused per step (mL)**	**Dose per step (mg)**	**Cumulative dose (mg)**
1	1	5	15	1.25	0.3	0.3
2	1	10	15	2.5	0.6	0.9
3	1	20	15	5	1.2	2.1
4	1	40	15	10	2.4	4.5
5	2	10	15	2.5	5.96	10.46
6	2	20	15	5	11.91	22.37
7	2	40	15	10	23.82	46.19
8	2	80	174.38	232.5	553.81	600
Total time (min) = 279.38 = 4.66 h						

Solution 1 is a tenfold dilution of the final target concentration. The concentration of solution 2 is calculated by subtracting the cumulative dose administered in steps 1–4 from the total target dose and dividing by the bag volume. Values shown are rounded to the nearest 2 decimal places

Depending on risk stratification, protocols with different number of dilutions can be utilized. Studies have found that patients with an initial negative carboplatin skin test result can tolerate a “rapid” 2-bag, 8-step protocol (Table 6) [4, 10, 36, 37]. For high-risk patients with initial severe reactions or breakthrough reactions during intermediate desensitization, a “prolonged” 4-bag, 16-step protocol can be used (Table 7) [52]. Similar rapid, intermediate, and prolonged protocols have been published for oxaliplatin [5, 10, 14].

**Table 7:** Sixteen-step desensitization protocol for carboplatin total dose 600 mg

	**Volume (mL)**	**Concentration (mg/mL)**	**Amount infused (mL)**	**Total mg per bag**		
Solution 1	250	0.0024	9.38	0.6		
Solution 2	250	0.024	9.38	6		
Solution 3	250	0.24	18.75	60		
Solution 4	250	2.38	250	595.25		
**Step**	**Solution**	**Rate (mL/h)**	**Time (min)**	**Volume infused per step (mL)**	**Dose per step (mg)**	**Cumulative dose (mg)**
1	1	2.5	15	0.63	0.0015	0.0015
2	1	5	15	1.25	0.003	0.0045
3	1	10	15	2.5	0.006	0.011
4	1	20	15	5	0.012	0.023
5	2	2.5	15	0.63	0.015	0.038
6	2	5	15	1.25	0.03	0.068
7	2	10	15	2.5	0.06	0.13
8	2	20	15	5	0.12	0.25
9	3	5	15	1.25	0.3	0.55
10	3	10	15	2.5	0.6	1.15
11	3	20	15	5	1.2	2.35
12	3	40	15	10	2.4	4.75
13	4	10	15	2.5	5.95	10.70
14	4	20	15	5	11.91	22.61
15	4	40	15	10	23.81	46.42
16	4	80	174.38	232.5	553.58	600
Total time (min) = 399.38 = 6.66 h						

Solution 1 is a 1000-fold dilution of the final target concentration; solution 2 is a 100-fold dilution of the final target concentration; solution 3 is a tenfold dilution of the final target concentration, and the concentration of solution 4 is calculated by subtracting the cumulative dose administered in steps 1–12 from the total target dose and dividing by the bag volume. Values shown are rounded to the nearest 2 decimal places. Note for Tables 5, 6, and 7: the total volume and dose dispensed are more than the final dose given to the patient because many of the solutions are not completely infused

In the largest published report to date of 2177 desensitizations to chemotherapy and monoclonal antibodies, 1069 carboplatin desensitizations were conducted using these protocols, of which 68% had no breakthrough HSR, 24% were mild, 4% were moderate, and 4% were severe without any deaths and with all patients being able to complete their treatment [31]. In other published studies, the percentage of patients experiencing a breakthrough HSR during rapid and intermediate protocols for carboplatin and oxaliplatin ranges from 35 to 59% (Table 8), with higher rates in ST-positive (37–79%) and ST converter (34–83%) patients compared to ST-negative patients (9–55%) [4, 36, 37, 53, 54]. As most breakthrough HSRs are mild, 97–100% of desensitizations in these studies were able to be completed, with less than 1–2% resulting in severe HSRs.

**Table 8:** Breakthrough reactions in patients undergoing platin desensitization

**Study**	**Agent**	**No. of steps in desensitization**	**No. of desensitizations completed**	**No. of patients with BTR**	**Severe BTR, n (% desensitizations)**	**No. of patients unable to complete treatment due to BTR**
Lee et al. (2004) [51]	Carboplatin	12	35	4/10 (40%)	0	0
Lee et al. (2005) [53]	Carboplatin	12	127	11/31 (35%)	1 (0.8%)	0
Hesterberg et al. (2009) [4]	Carboplatin	8 or 10	105	13/30 (43%)	1 (0.9%)	1
Patil et al. (2012) [36]	Carboplatin	8 or 12	148	23/39 (59%)	0	0
Wong et al. (2014) [14]	Oxaliplatin	8 or 13	200	17/48 (35%)	1 (0.5%)	0
Sloane et. al. (2016) [31]	Carboplatin Cisplatin Oxaliplatin	12–16^a^	Carboplatin 1069	NR; 253 (24%) carboplatin desensitizations had mild HSR and 87 (8%) had moderate–severe HSR	Carboplatin 41 (4%)	0
Mawhirt et al. (2018) [54]	Carboplatin Oxaliplatin	12	146^b^	21/36 (58%)^b^	3 (2%)^b^	NR

*BTR* breakthrough reaction, *NR* not reported

^a^A total of 22 desensitizations were performed that were < 12 steps

^b^Data is combined for carboplatin and oxaliplatin as results of desensitizations were not distinguished

### Taxanes

Similar rapid (2-bag, 8-step), intermediate (3-bag, 12-step), and prolonged (4-bag, 16-step) desensitization protocols have been published for taxanes [10, 27, 32]. The successful use of a 12-step desensitization for paclitaxel and docetaxel was first published in a series of 17 gynecologic oncology patients who underwent 77 desensitizations [20]. In the large desensitization study by Sloane et al. referenced above, paclitaxel was the drug with the lowest rate of reactions (15% of desensitizations), and only 2% of breakthrough HSRs were severe [31]. The percentage of patients experiencing an immediate breakthrough HSR in other studies has been reported at 21–30% (Table 9), and one also observed delayed breakthrough HSRs in 14% of patients [27, 32, 53]. Atopy, but not initial HSR severity or ST result, was found to be associated with increased risk of immediate HSR during desensitization or challenge [32].

**Table 9:** Breakthrough reactions in patients undergoing taxane desensitization

**Study**	**Agent**	**No. of steps in desensitization**	**No. of desensitizations completed**	**No. of patients with BTR**	**Severe BTR**	**No. of patients unable to complete treatment due to BTR**
Lee et al. (2005) [53]	Paclitaxel Docetaxel	12	Paclitaxel 114 Docetaxel 2	6/22 (27%) 0	NR	0
Picard et al. (2016) [32]	Paclitaxel Docetaxel	8–16	940	29/138 (21%)	0	0^a^
Otani et al. (2018) [27]	Paclitaxel	8–17	NR	9/30^b^ (30%)	0	0

*BTR* breakthrough reaction, *NR* not reported

^a^One patient stopped treatment because of adverse reaction with paclitaxel-induced pneumonitis

^b^Five of 35 patients in the study were rechallenged without desensitization

As can be seen, there are a number of different desensitization protocols that are variations on the theme of gradual uptitration from very low starting doses. These protocols can be further individualized if a patient experiences breakthrough HSRs by adding or modifying steps or premedications. Protocols utilized may also depend on the equipment and resources available at each institution.

Of note, premedications vary significantly between desensitization protocols, and there are limited data available on optimal premedication regimens. Different protocols may incorporate H1 antihistamines, H2 blockers, steroids, montelukast, and/or aspirin and other COX-1 inhibitors. These differences must also be taken into account when evaluating desensitization outcomes.

### One-Bag Protocols

In addition to these multi-bag desensitization protocols, one-bag desensitization protocols have been published, although their use has not been validated. At some institutions, a one-bag protocol at standard concentration may be chosen for feasibility of preparation or in response to concerns about stability at lower dilutions [28]. Protocols ranging from 9 to 17 steps have been described for platins and taxanes in small patient cohorts (*n* = 30–49 for platins, *n* = 24–25 for taxanes) [28, 55–58]. Again, the severity of initial HSRs is an important factor to consider, as the percentage of initial grade 3 HSRs varied from 10 to 25% for platins [55, 56, 58] and 16 to 38% for taxanes [55, 57] in these studies. The percentage of patients experiencing breakthrough HSR with these one-bag protocols ranges from 27 to 61% for platins (with up to 6–8% requiring epinephrine or not being able to complete desensitization) [55, 58] and lower for taxanes.

Comparison of study populations and outcomes for different desensitization protocols is shown in Table 10. The 3-bag 12-step and 4-bag 16-step protocols are the most widely used, having been validated in over 3000 published cases and shown to be effective for chemotherapy agents, monoclonal antibodies, and antibiotics, even in severe HSRs [31, 41, 59]. Grade 3 breakthrough HSRs are extremely rare, occurring in less than 1% of cases, and over 99% of desensitizations can be completed despite breakthrough HSRs [5, 31, 32, 41, 60]. Two-bag protocols have been developed for patients with initial mild-to-moderate reactions and negative ST, or patients who have tolerated standard 3-bag protocols [4, 14, 32, 36, 60]. One-bag protocols have garnered increasing interest due to ease of preparation and administration, but thus far, studies are limited to smaller numbers of patients and study populations may not be equivalent, especially for more severe initial HSRs.

**Table 10:** Comparison of desensitization protocols

**Protocol**	**Study characteristics**	**Outcomes**	**Considerations**
3-bag 12–13 step 4-bag 16–17 step	> 3000 published desensitizations **Agents** - Carboplatin, cisplatin, oxaliplatin [5, 14, 31, 36, 41, 54] - Paclitaxel, docetaxel [27, 31, 32, 41] - Monoclonal antibodies (e.g., rituximab, infliximab) [31, 41, 60] **Initial HSR severity** (Brown) [5, 31, 32] - Grade 1 10–32% - Grade 2 16–73% - Grade 3 17–52%	**Breakthrough HSRs** - 26% of desensitizations [5, 31] - Grade 2, 7%* [31, 32, 60] - Grade 3, < 1%* [31, 32, 60] **Completed** > 99% [14, 31, 32, 36, 41]	The 3-bag and 4-bag protocols are the most widely used and can be considered for most patients, particularly those with initial severe HSR and platin HSR [31, 41] Published risk stratification pathways used initial HSR severity, ST results, and patient characteristics to risk stratify patients to each protocol [31, 32]
2-bag 8-step	> 150 published desensitizations **Agents** - Carboplatin, oxaliplatin [4, 14, 36] - Paclitaxel, docetaxel [27, 32] **Initial HSR severity**: NR	**Breakthrough HSRs** - 12% of desensitizations [14] - Severity NR **Completed** > 99% [4, 14, 36]	Published risk stratification pathways used initial HSR severity, ST results, and time since last reaction (for platins) to risk stratify patients to this protocol [14, 27, 32, 36] Criteria for receiving 2-bag 8-step protocols in published studies were: - History of platin HSR > 1 month prior and negative ST [14, 36] - History of previously tolerating 3-bag protocol (above) for taxanes without breakthrough reaction [32]
1-bag 9-step	490 published desensitizations **Agents** - Carboplatin, cisplatin, oxaliplatin [55] - Paclitaxel, docetaxel [55] - Monoclonal antibodies (e.g. rituximab, cetuximab) [55] **Initial HSR severity** (Brown) [55] - Grade 1 35% - Grade 2 53% - Grade 3 12%	**Breakthrough HSRs** - 5% of desensitizations (88% with platins) - Grade 2, 2% - Authors reported that none of the breakthrough HSRs were grade 3, but epinephrine was used for 3 of the grade 2 breakthrough HSRs **Completed** > 99% [55]	Most patients who received this protocol (*n* = 90) had less severe initial HSR than other studies For patients with mild initial HSR and negative ST, ease of preparation and shorter duration could make this an option at centers preferring to perform desensitization in the outpatient setting
1-bag 12–13 step	299 published desensitizations **Agents** - Oxaliplatin, carboplatin, cisplatin [56] - Paclitaxel [57] **Initial HSR Severity** (Brown) [56, 57] - Grade 1 4–14% - Grade 2 58–61% - Grade 3 25–38%	**Breakthrough HSRs** - 16–17% of desensitizations - Grade 2, 7–8% - Grade 3, 1% **Completed** > 98% [56, 57]	Use of this protocol required a high-precision pump to deliver low doses

*NR* not reported

^*^Applies to 3-bag 12-step protocol which constituted the majority of desensitizations. For 4-bag 16-step protocols, the rate of reactions is lower based on author’s clinical experience

### Four-Step Reintroduction Protocols

Four-step reintroduction protocols for administration of platins and taxanes have been published, variably labeled as “desensitization” or “graded challenge,” and have also not been validated in large studies. These are reminiscent of but distinct from prior reports of extended infusions. Two centers previously studied the use of prophylactic extended carboplatin infusion in patients with recurrent ovarian cancer and no prior history of HSR. They found that the administration of 1%, 10%, and 90% of the total dose, respectively, in three 60-min steps did not decrease rates of subsequent HSR relative to standard infusion [61, 62].

More recent studies using 4-step protocols have raised interest in whether risk stratification based on initial HSR severity and ST results can identify patients who can tolerate shorter protocols. One study that used ST after HSRs identified 21 platinum ST-negative and 12 taxane ST-negative patients who were able to tolerate the same agent via 4-step challenge, as well as others who could tolerate an alternate agent in the same class [39]. However, there are important differences to note about the patient populations studied in 4-step desensitization protocols, especially for platins. The protocol reported by Li et al. for carboplatin and cisplatin was restricted to patients with mild to moderate low-risk initial HSRs. A later publication found that, when applied to patients with initial moderate (high-risk) HSRs, 4/20 (20%) experienced severe breakthrough HSRs that prevented treatment completion [58, 63]. The rapid 4-bag, 4-step protocol published by Altwerger et al. included 73/129 (57%) patients with no prior history of HSR but who had positive skin test results when screened after 6 cycles of carboplatin, which may represent a different population [64]. In addition, there was one death reported in this study, in a patient with underlying pulmonary hypertension who had tolerated multiple previous desensitizations. As a result, careful consideration of risk profiles is needed to determine if and when such protocols can be safely used.

## Pathways

Institutions have developed different pathways for chemotherapy HSR management that incorporate the elements in this review, including grading of reaction severity, skin testing, drug challenge, and desensitization.

The choice of setting and available resources, including nursing-to-patient ratio, may also affect an institution’s pathway and the ability to perform certain procedures such as skin testing. Depending on the resources available, desensitizations may occur in the ICU, inpatient acute care ward, outpatient infusion center, or outpatient specialized allergy unit.

Different approaches have been published around the safe reintroduction of platinum agents following HSR. These approaches include use of ST to determine the initial desensitization protocol, with or without serial ST in patients with remote HSR to identify ST conversion as described above [14, 36, 37, 39]. However, routine skin testing can be challenging due to varying institutional regulations and resources, as well as burdensome for patients who are chronically ill and already require frequent healthcare encounters. A more widely acceptable and feasible pathway may be to use a 12-step protocol for patients with initial mild or moderate HSRs and a 16-step protocol for patients with initial severe HSRs [5, 31]. In addition, as the sensitivity of ST for certain agents such as oxaliplatin is not known, desensitization can be offered to patients with or without a positive ST result, regardless of initial HSR severity [5]. For patients with no breakthrough HSR, the same or potentially shorter protocol could be used. For patients who experience breakthrough HSR, the severity of the reaction can guide individualized adjustments to the dilutions, steps, and premedications, or decisions to use a longer protocol.

**Fig. 2:**
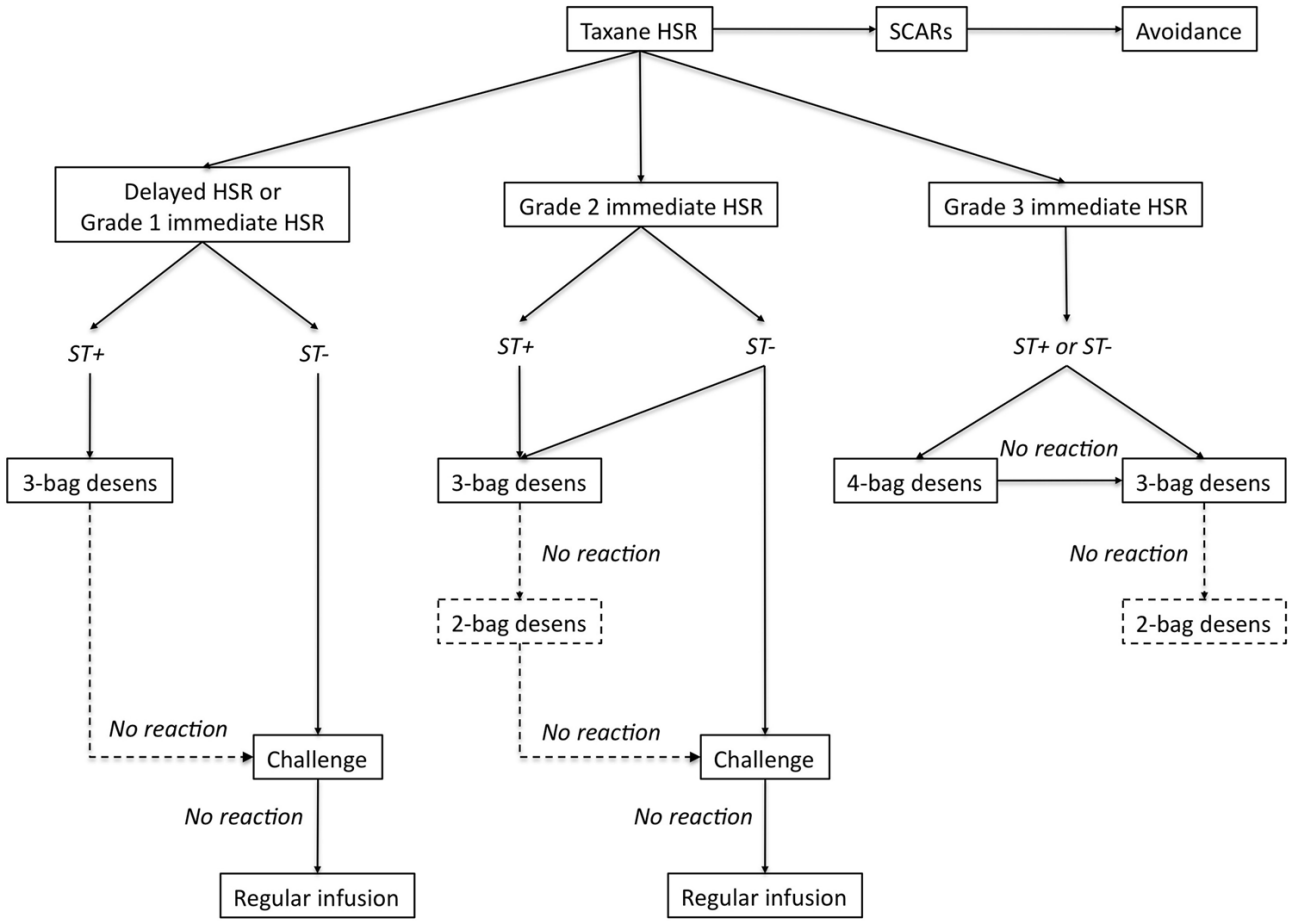
Approach to reintroduction of taxanes after HSRs. Patients with a history of severe cutaneous adverse drug reactions (SCARs) including Stevens-Johnson syndrome and blistering skin reactions should avoid taxanes. Grading of immediate HSR severity is based on Brown’s classification. Patients with delayed or grade 1 immediate HSRs with negative skin testing (ST −) can undergo challenge. The decision to perform desensitization or challenge in patients with grade 2 immediate HSRs who are ST − is based on patient comorbidities and comfort with the procedure. Patients with grade 3 HSRs, regardless of ST result, undergo desensitization. Institutions that do not have access to ST can follow the protocol for ST + patients. Patients who do not have breakthrough HSRs during the initial protocol can subsequently be treated with a shorter desensitization protocol, challenge, or regular infusion according to the algorithm. For patients who experience breakthrough HSRs, adjustments can be made to premedications and length of protocol. Reproduced from Fig. 1 in Picard et al. [32] with permission

Similarly, for taxane HSRs, the approach to reintroduction relies on characteristics and grade of initial HSR, as well as ST results if available (Fig. 2) [32]. Challenge can be considered for patients with grade 1 HSRs and negative ST, while patients with grade 2 to 3 immediate HSRs and positive ST undergo desensitization. As with platinum agents, the severity of breakthrough HSRs during desensitization or challenge can guide individualized modifications or decisions to utilize a slower protocol. Patients without breakthrough HSR can advance toward a shorter protocol, challenge, or regular infusion. When ST is not available, institutions can follow the pathway as if patients are ST-positive, which enables a more cautious approach, or use grading of the initial HSR severity alone to risk-stratify patients into desensitization protocols of different lengths versus challenge [27].

## Discussion

As this review shows, the optimal use of desensitization protocols in different patient populations and settings is still being tailored. With increasing safety data over time, more patients with mild-to-moderate initial HSRs, and even some with severe initial HSRs, have been desensitized in outpatient infusion centers at institutions experienced with RDD, instead of in the ICU [31]. This has significant advantages in terms of patient access and convenience, ease of scheduling, and resource demands. As pharmacy and nursing resources differ between inpatient and outpatient settings, reports of ongoing experiences in the outpatient setting will add to the literature on safety and efficacy.

The information outlined here is intended to help institutions determine what pathway might best meet their needs. These decisions require consideration of the time and resources required for desensitization, urgency of treatment, and availability of trained staff and standardized protocols for treatment of HSRs. Institution-specific factors may also affect decisions around use of skin testing, drug challenge, and choice of desensitization protocol. For example, serial ST may be more beneficial in situations when advancing from slower to faster desensitization infusions would improve convenience or quality of life for patients—by enabling transition to outpatient slowed infusion at centers where desensitization must occur in the ICU. In contrast, if patients can receive empiric desensitizations in the outpatient infusion center, additional visits for skin testing may not be desired by the patient, even if feasible. As discussed above, institutions differ in terms of whether drug challenge is even considered in the initial evaluation for low-risk or ST-negative patients, or only after uneventful desensitizations.

## Summary and Conclusions

The last two decades have witnessed significant advances in our understanding of risk factors, clinical phenotypes, and mechanisms of chemotherapy HSRs, as well as the role of diagnostic tools like skin testing. Desensitization to platinum and taxane agents has been shown to be safe and effective. In addition, RDD seems to be comparable to standard infusion in terms of treatment efficacy. In the largest desensitization study to date, patients with recurrent ovarian cancer receiving carboplatin via desensitization had a nonsignificant trend toward increased life expectancy compared to matched controls receiving standard infusion, suggesting that at the least there is no trade-off in terms of mortality [31]. While drug desensitization has been applied to an increasing number of chemotherapy agents and biologics, there is ongoing interest in the development of better endophenotyping tools and biomarkers to personalize treatment.

Regardless of the pathway chosen, the optimal evaluation and management of patients with chemotherapy HSRs relies on multidisciplinary collaborations between allergy, oncology, pharmacy, nursing, and in some institutions critical care. Chemotherapy desensitizations and drug challenges are high-risk, high-complexity procedures that should be carried out with expert allergy guidance in settings equipped to treat reactions. Standardizing classification systems and research methods will increase knowledge of safe and personalized management strategies for patients and enable institutions to adapt these pathways to their unique needs.
